# Early experience with pericardiectomy for constrictive pericarditis in Ile-Ife, Nigeria: a retrospective analysis

**DOI:** 10.4314/ahs.v25i4.17

**Published:** 2025-12

**Authors:** Olugbenga O Ojo, Uvie U Onakpoya, Oluwaseun R Akanbi, Abayomi E Oguns, Mathias O Ikokoh

**Affiliations:** 1 Department of Surgery, Obafemi Awolowo University/Obafemi Awolowo University Teaching Hospitals Complex, Ile-Ife; 2 Department of Surgery, Obafemi Awolowo University Teaching Hospitals Complex, Ile-Ife; 3 Department of Anaesthesia and intensive care, Obafemi Awolowo University Teaching Hospitals Complex, Ile-Ife

**Keywords:** constrictive pericarditis, pericardiectomy, diastolic heart failure, New York heart association (NYHA)

## Abstract

**Background:**

Constrictive pericarditis is a rare but important cause of diastolic heart failure. Its uniqueness lies in the fact that surgery (pericardiectomy) remains the mainstay of treatment. Globally, the leading causes of constrictive pericarditis include idiopathic, mediastinal irradiation, post cardiac surgery and tuberculosis.

**Methods:**

Patients who underwent pericardiectomy at our tertiary hospital between January 2019 and December 2024 were retrospectively studied with the aim of discussing our experience with the procedure and its outcomes. Data including baseline demographics, preoperative conditions, intraoperative details, and postoperative outcomes were collected from clinical records and analysed.

**Results:**

Thirteen patients had total pericardiectomy during the period under review. The median age was 28 years with dyspnoea and ascites being the most common symptoms. Most patients (61.6%) presented in NYHA class III and IV and were above ASA II classification at the time of surgery. Pericardiectomy was done via median sternotomy and without cardiopulmonary bypass in all cases, with an average surgery duration of 284.5 mins. Postoperative complications included low cardiac output, acute kidney injury, coagulopathy, and prolonged pleural effusion. Median duration of intensive care unit (ICU) stay was 2days and there was 1 mortality. At twelve months follow up, more than 90% of surviving patients were in NYHA class I or II.

**Conclusion:**

Pericardiectomy offers symptomatic relief to patients with constrictive pericarditis. Early identification of this disease would prevent disease progression and offer improved outcomes.

## Introduction

Constrictive pericarditis (CP) is one of the surgically correctable causes of heart failure. It results from chronic inflammation and fibrosis of the pericardium leading to impairment of diastolic filling and right heart failure^1^,^2^. The aetiology of constrictive pericarditis is diverse, with viral or idiopathic causes and post cardiotomy irritation being the leading causes in Western population. In developing countries, tuberculosis remains the main aetiology for chronic inflammation leading to constrictive pericarditis^3^.

Early diagnosis of CP and referral for pericardiectomy has been shown to improve outcomes and chances of achieving post operative normal cardiac functions^4^,^5^. Though pericardiectomy with its various modifications and surgical approaches remains the mainstay of treatment for constrictive pericarditis, several reports show that post operative hemodynamics have varied from normal findings making several researchers to arrive at the conclusion that restoration of normal cardiac function rarely occurs after a pericardiectomy^6^–^8^.

Although medical therapy for heart failure with or without treatment for tuberculosis is usually commenced by the referring physicians, this should not necessarily delay the referral and performance of a pericardiectomy in patients who are fit. The aim of this study is to review the early experience with pericardiectomy in the treatment of constrictive pericarditis in Ile-Ife.

## Materials and Methods

All patients who had clinical, echocardiographic, radiological and pathological post-resection confirmation of constrictive pericarditis and who underwent pericardiectomy for constrictive pericarditis at the Obafemi Awolowo University Teaching Hospitals Complex (OAUTHC) Ile-Ife between January 2019 and December 2024 were retrospectively reviewed. Data was obtained from hospital medical records using a predesigned proforma. Patients who had concomitant pericardiectomy while undergoing repair of congenital or acquired heart diseases were excluded. The procedure performed in all patients was a total pericardiectomy, from phrenic to phrenic and from great vessels to diaphragm ensuring that, the caval vessels were also free of constriction.

The data included, demographic characteristics, presenting complaints, preoperative functional status, intraoperative details, and postoperative outcome. In-hospital mortality was defined as death occurring within 30 days of operation or within the hospitalization period of the operation.

### Statistical analysis

Data obtained was analysed with the SPSS statistical package (version 27). Categorical variables were expressed as percentages and proportions while continuous variables were expressed as mean and standard deviations. Association between variables were analysed by the fisher exact test for categorical variables and the student's t-test for continuous variables. A p-value of ≤0.05 was considered statistically significant.

Ethical approval was obtained from the ethics and research committee of the Obafemi Awolowo University Teaching Hospital (OAUTHC) Ile-Ife.

## Results

Thirteen patients had pericardiectomy during the period comprising 7 males (53.8%) and 6 females (46.2%) with a male/female ratio of 1/1 in the study population. The median age was 28 years (IQR:18-39;) and three (23.1%) patients were less than 18 years old. The most common presenting complaints were dyspnoea and ascites (Table 1). Most patients (61.6%) presented in New York Heart Association (NYHA) functional class III or IV and were above ASA II classification at the time of surgery. The median duration of symptoms before diagnosis was 8 months (IQR: 2.5-30) and all patients had echocardiography done while 8 patients (61.5%) had computerized tomography scan in addition to echocardiographic diagnosis. Six cases (46.2%) were effusive constrictive pericarditis and seven (53.8%) were non-effusive (fibrous constrictive) pericarditis. All cases were done without the use of cardiopulmonary bypass, and the median duration of surgery was 302minutes (IQR: 229-330). The, median post operative blood loss was 450 mL (IQR:300-1250) and only one patient required re-sternotomy due to excessive mediastinal bleeding. All patients were extubated on table and transferred to the intensive care unit (ICU) with a median ICU stay of 2days:(IQR: 2-2) (Table 2).

**Table 1:** Patients baseline/pre-operative characteristics (n=13)

Characteristic/Variable	Value n (%)
**Age**	
Median	28 years
**IQR	18 years
<18years	3 (23.1%)
>18years	10(76.9%)
**Gender**	
Male	7(53.8%)
Female	6(46.2%)
**Symptomatology**	
Dyspnoea	13(100%)
Orthopnoea	8(61.5%)
Jugular venous distension	10(76.9%)
Hepatomegaly	9(69.2%)
Ascites	12(92.3%)
Right pleural effusion	4(30.8%)
Bilateral pleural effusion	6(46.2%)
**Diagnostic modality**	
Echo alone	5(38.5%)
Echo + chest CT	8(61.5%)
	
**Diagnostic delay (days)**	
No < 14days	9(69.2%)
Yes > 14days	4(30.8%)
	
**Type of Constrictive pericarditis**	
Fibrous constrictive	7(53.8%)
Effusive constrictive	6(46.2%)

* CT= computerized tomography

** IQR = interquartile range

**Table 2:** Perioperative and post-operative characteristics

Characteristic/Variable	Value n (%)
**Duration of Surgery**(mins)	
Median	302
*IQR	229.5 to 330 mins
**Estimated blood loss**(mls)	
Median	450
IQR	300 to 1,250 mins
**Intensive Care Unit (ICU) Stay**(days)	
Median	2 days
IQR	2 to 21
	
**Inotrope**	
Yes	3(23.1)
No	10(76.9)
	
**Complications**	
*LCOS	4(30.7%)
Coagulopathy	1(7.7%)
Inadvertent cardiotomy	1(7.7%)
Prolonged pleural effusion	1(7.7%)
	
**Histology of resected pericardium**	
Tuberculous pericarditis	5(38.5%)
Nonspecific chronic inflammation (Probably Tuberculous)	8(61.5%)

* IQR= interquartile range, LCOS=Low cardiac output syndrome, minutes(mins), millilitres(mls)

Low cardiac output state was the commonest complication seen in this study occurring in 4(30.8%) patients with 3 of them requiring post operative inotropic support (Table 2). There was one in-hospital mortality in a patient with COVID-19 infection who developed a coagulopathy on post operative day 1. At 12 months follow-up period 4 (33.3%) out of the surviving patients were in NYHA functional class I, while 8 (66.7%) patients were in NYHA class II. Histopathological examination of specimen taken at pericardiectomy confirmed tuberculous pericarditis as the aetiology in 5(38.5%) and nonspecific chronic inflammation possibly due to tuberculosis in the remaining 8(61.5%) cases.

## Discussion

Constrictive pericarditis is a relatively rare but notable cause of diastolic heart failure. Even in centres where large series have been published, there are fewer than twelve cases annually^9^–^13^.

This study presents early findings from a major referral tertiary institution in southwest Nigeria and demonstrates that many patients in this environment present late, with 61.6% of them being classified as New York Heart Association (NYHA) functional class III and IV upon presentation. Our finding is contrary to other studies^9^,^11^,^14^ where most patients were in NYHA class II or III. Biçer et al. found 76% of patients and Peset et al. found 67% in these classes^14^,^15^. The difference in NYHA functional class at presentation between our study and previous studies may be attributed to variations in health-seeking behaviour among patients in different environments. Similar studies from Kumasi and Accra in Ghana also found that most patients presented in NYHA functional class III and IV^16^,^17^. Dyspnoea was the most prevalent symptom observed in our study, consistent with the findings of Biçer et al.

The median age in this study was 28 years, with an interquartile age range of 18 to 39 years which is similar to other publications in the West African subregion^16^,^17^ In the study conducted by Tettey et al^16^., the average age of participants was 33 years and,. Lin et al^11^,. also demonstrated comparable findings in their research.

Diagnosis of constrictive pericarditis was made in less than 14 days in 69.2% of the patients. Constrictive pericarditis was diagnosed using transthoracic echocardiography in 5 patients (38.5%) and a combination of echocardiography and chest CT scan in 8 patients (61.5%).

Consistent with the findings of numerous studies conducted in developing countries,^7^,^16^,^17^,^18^ this study identified tuberculosis as the most prevalent cause of constrictive pericarditis in treated patients.

**Figure 1:**
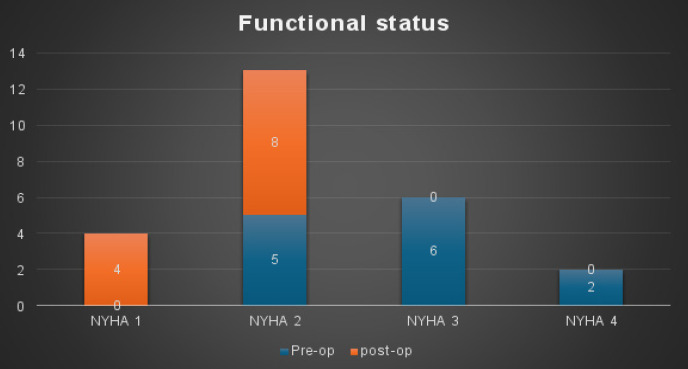
Pre and post-operative functional status

All the pericardiectomies in this study were done via a full median sternotomy without the use of cardiopulmonary bypass and NYHA functional status improved to at least NYHA class II. Figure 1.

Chowdhury et al.,^7^ found that total pericardiectomy (phrenic to phrenic) via a median sternotomy resulted in early normalization of hemodynamic parameters and improved long-term survival, without the routine use of cardiopulmonary bypass being necessary. Similarly, Biçer et al., demonstrated an improvement in functional status by at least one NYHA class in 80% of their patients^14^.

Low cardiac output syndrome (LCOS) was the most common complication, consistent with previous studies^7^,^16^,^18^. However, three out of the four patients with postoperative LCOS needed inotropic support, and all showed improved cardiac function within a week. No mortality was observed among patients with postoperative low cardiac output syndrome (LCOS), and all exhibited improvement in their functional status.

In-hospital mortality occurred in 1 (7.7%) patient, aligning with studies reporting 6-10% mortality rates. More recent studies by Welch et al. and Biçer et al. found rates below 5%^4^,^14^.

A larger study from sub-Saharan Africa reported a 16% in-hospital mortality rate due to procedures performed in an earlier decade, with low output cardiac failure being the major cause.^18^ In our study, in-hospital mortality occurred in a patient with COVID-19 infection who was operated upon within 2 weeks of a negative PCR (polymerase chain reaction) test result and who experienced significant mediastinal bleeding and required re-sternotomy, where no surgical bleed was found. The haemorrhage and subsequent demise were attributed to disseminated intravascular coagulopathy likely caused by COVID-19 infection.

At a twelve-month follow-up, over 90% of surviving patients were classified as NYHA class I or II. In agreement with the findings of Tettey et al.,^16^ it is believed that this outcome can generally be achieved by ensuring the adequate removal of the thickened pericardium over the right atrium and the superior and inferior vena cavae as part of the procedure. Chowdhury et al.,^7^ have demonstrated that this can only be accomplished via a full median sternotomy with or without cardiopulmonary bypass.

## Conclusion

Our initial experience with pericardiectomy for constrictive pericarditis shows promising results, comparable to larger studies globally and demonstrates that total pericardiectomy can safely be performed without cardiopulmonary bypass in most cases.
